# Usability of Ultrasonic Frequency Testing for Rapid Generation of High and Very High Cycle Fatigue Data

**DOI:** 10.3390/ma14092245

**Published:** 2021-04-27

**Authors:** Michael Fitzka, Bernd M. Schönbauer, Robert K. Rhein, Niloofar Sanaei, Shahab Zekriardehani, Srinivasan Arjun Tekalur, Jason W. Carroll, Herwig Mayer

**Affiliations:** 1 Department of Material Sciences and Process Engineering, Institute of Physics and Materials Science, University of Natural Resources and Life Sciences (BOKU), 1190 Vienna, Austria; michael.fitzka@boku.ac.at (M.F.); bernd.schoenbauer@boku.ac.at (B.M.S.); 2 Center for Materials and Manufacturing, Eaton Corporation, Southfield, MI 48076, USA; RobKRhein@eaton.com (R.K.R.); NiloofarSanaei@eaton.com (N.S.); ShahabZekriardehani@eaton.com (S.Z.); SrinivasanArjunTekalur@Eaton.com (S.A.T.); JasonWCarroll@eaton.com (J.W.C.)

**Keywords:** ultrasonic fatigue, frequency effect, strain rate effect, environmental effect, size effect, high cycle fatigue, very high cycle fatigue

## Abstract

Ultrasonic fatigue testing is an increasingly used method to study the high cycle fatigue (HCF) and very high cycle fatigue (VHCF) properties of materials. Specimens are cycled at an ultrasonic frequency, which leads to a drastic reduction of testing times. This work focused on summarising the current understanding, based on literature data and original work, whether and how fatigue properties measured with ultrasonic and conventional equipment are comparable. Aluminium alloys are not strain-rate sensitive. A weaker influence of air humidity at ultrasonic frequencies may lead to prolonged lifetimes in some alloys, and tests in high humidity or distilled water can better approximate environmental conditions at low frequencies. High-strength steels are insensitive to the cycling frequency. Strain rate sensitivity of ferrite causes prolonged lifetimes in those steels that show crack initiation in the ferritic phase. Austenitic stainless steels are less prone to frequency effects. Fatigue properties of titanium alloys and nickel alloys are insensitive to testing frequency. Limited data for magnesium alloys and graphite suggest no frequency influence. Ultrasonic fatigue tests of a glass fibre-reinforced polymer delivered comparable lifetimes to servo-hydraulic tests, suggesting that high-frequency testing is, in principle, applicable to fibre-reinforced polymer composites. The use of equipment with closed-loop control of vibration amplitude and resonance frequency is strongly advised since this guarantees high accuracy and reproducibility of ultrasonic tests. Pulsed loading and appropriate cooling serve to avoid specimen heating.

## 1. Introduction

Ultrasonic fatigue testing is a powerful method to investigate the fatigue properties of materials in high cycle fatigue (HCF) and very high cycle fatigue (VHCF) regimes. Specimens are stimulated by resonance vibrations at a frequency close to 20,000 Hz rather than being stressed by external forces as in conventional (e.g., servo-hydraulic, rotating bending, resonance tester) fatigue tests. The high cycling frequency shortens testing times and makes fatigue investigations at extremely high numbers of cycles possible, in a regime that is hardly accessible with conventional testing due to excessive testing times. Thus, ultrasonic fatigue testing is the most appropriate technique to study fatigue properties of materials in the VHCF regime [1].

One of the main questions involved with ultrasonic testing is whether the measured fatigue data would be similarly obtained using conventional testing methods working at much lower frequencies [2,3,4,5]. Fatigue tests with servo-hydraulic test frames, resonance testing apparatus or rotating bending equipment are typically performed at cycling frequencies below 100 Hz. Moreover, technical components are typically stressed at cycling frequencies far below the ultrasonic range, so that cycling frequencies in conventional tests are much more comparable to those prevailing in actual applications. It is important for high-frequency testing to consider possible frequency effects on the progress of fatigue damage and the measured fatigue lifetimes. Additionally, there are generally accepted standards for how to perform servo-hydraulic tests (i.e., measurement and control of the applied forces) or rotating bending tests (i.e., application of a defined bending moment). Although there have been individual attempts (e.g., WES 1112 [6] requiring control of displacement amplitude), a similar degree of standardisation is not available for ultrasonic tests. Different setups for ultrasonic fatigue testing are being used, which influence the accuracy and replicability of tests and the measured data.

This paper gives an overview of the existing knowledge on the applicability of ultrasonic fatigue testing for material qualification and collection of fatigue data based on the experimental data generated by the authors, as well as data gathered from the literature. An ultrasonic testing setup is described that enables reproducible and high-precision fatigue tests, avoids specimen heating, and controls, monitors and logs specimen loading. It was shown that extrinsic influences, such as size effects can influence the measured cyclic properties. The effect of a high strain rate on plastic deformation and crack initiation [7,8] and the time-dependent influence of the testing environment [9,10] are considered as the two, in principle, possible influences of the testing frequency on fatigue damage.

Fatigue performance of aluminium alloys, steels and titanium alloys as well as nickel alloys, magnesium alloys, fibre-reinforced polymer composites and graphite measured with conventional and ultrasonic equipment were compared based on the data available in the literature. Moreover, original fatigue data generated by the authors on a Ti6Al4V and 2024-T351 aluminium alloy by both conventional and ultrasonic fatigue were used in this study for discussion and comparison purposes. The present work aims to review and compare ultrasonic and conventional fatigue data, discuss the potential variations between these results, and offer insights into how these data correlate for each material.

## 2. Materials and Methods

### 2.1. Ultrasonic Fatigue Testing Procedure

In an ultrasonic test, an appropriately designed specimen is stimulated to resonance vibrations at close to 20 kHz. The displacement movement of the specimen is sinusoidal with time with maximum vibration amplitudes at both ends of the specimen. Cyclic loading is determined by the amplitude and frequency of the ultrasonic vibration. Strain amplitudes are highest in the centre of the specimen, where a vibration node is formed. Strain gauges are commonly used to measure the cyclic strain amplitude and to calibrate the ultrasonic fatigue test. Alternatively, several non-contact methods to measure strains at high frequencies have been employed in the past (e.g., optical interferometry based strain/displacement gauge [11], or laser vibrometry [12]). Particularly high temperatures may limit the applicability of strain gauges. They are also not suitable to be used for the actual control of the strain amplitude during a test. Cyclic stresses (or cyclic forces) cannot be measured directly but they can be calculated from the measured strains. In the high and very high cycle fatigue range, where ultrasonic fatigue testing is used, many materials show approximately linear elastic behaviour. Then, cyclic stresses, σ are calculated from the measured strains, ε with Young’s modulus, *E* and using Hooke’s law (Equation (1)).

*σ* = *E* × *ε* (1)

No widely accepted standard exists for how to perform ultrasonic fatigue tests. A successfully used method to control ultrasonic loading is to measure the vibration amplitude and to control it in a closed-loop [5]. The vibration amplitude of the specimen’s ends and strain amplitudes in the specimen’s centre are directly proportional. Therefore, the measured vibration amplitude is a suitable signal to control cyclic loading. Vibration amplitude can, for example, be measured with an induction coil, with a laser vibrometer or with a gap sensor. Strain gauges attached to the specimen’s centre also deliver a signal proportional to the cyclic load. However, strain gauges will fatigue and fracture during the high strains and high numbers of cycles that occur during an ultrasonic test and, therefore, are not suitable for control purposes.

By using the measured displacement signal in a closed-loop circuit, the vibration amplitude can be controlled with high accuracy. The maximum deviation between selected and actually realised vibration amplitudes can be limited to ±1%. In a second closed-loop circuit, the stimulation frequency is controlled and kept very close to the resonance frequency of the load train within ±1 Hz. Control of stimulation frequency is necessary, since the actual resonance frequency of the system can change during the test due to slight variations of temperature or due to the initiation of a crack, for example. The vibration amplitude can be measured and the magnitude and number of cycles applied can be stored using a computer unit for backtracking the specimen’s loading after fracture [5].

The measured fatigue data is strongly influenced by the chosen method of ultrasonic fatigue test. One of the most basic problems related to ultrasonic fatigue cycling is the heat generated in the specimen due to internal friction. Cyclic plastic deformation as well as the movement of interstitial atoms (i.e., Snoek effect in carbon steel) leads to heat dissipation and can increase the specimen’s temperature [13,14]. If specimens are loaded continuously at ultrasonic frequencies, their temperature may increase beyond acceptable values. For about 40 years, intermittent loading with periodic sequences of pulses and cooling pauses has been known as the appropriate method to avoid specimen heating [15]. Additionally, the specimen may be cooled with forced air. Both intermittent loading and forced air-cooling are appropriate methods to limit temperature increase by a maximum of 5 °C to 10 °C above room temperature during tests in ambient air, for example. Specimen temperature can be monitored with thermocouple or contact-free infrared thermography.

Intermittent loading requires precise closed-loop control of the vibration amplitude. Otherwise, overshoot of the amplitude at the beginning of the pulse can lead to undefined specimen loading and inaccurate tests. The rapid increase of the nominal vibration amplitude without overshoot cannot be guaranteed by all ultrasonic fatigue testing systems. Some simpler testing systems do not employ feedback control of cyclic displacement, but rather rely on controlling power output to the ultrasonic converter, which yields inferior loading amplitude accuracy. Measured ultrasonic fatigue data are influenced by the way the test is performed, the accuracy of specimen loading and the method of data evaluation. Therefore, differences between ultrasonic and conventional fatigue tests may not only be the result of intrinsic frequency influences but may also be a consequence of an improper experimental setup and an unsuitable testing procedure.

### 2.2. Servo-Hydraulic Fatigue Testing Procedure

In contrast to ultrasonic fatigue tests, testing with servo-hydraulic equipment is highly standardised regarding testing procedure, closed-loop control of static and dynamic force, data-acquisition systems and accuracy requirements. The choice of specimen geometry is not limited by the resonance criterion, also enabling investigations with specimens with large testing volumes and component testing.

Original fatigue data generated by the authors on Ti6Al4V and 2024-T351 aluminium alloys were used in this study. These tests were performed using MTS 810.10 servo-hydraulic fatigue testing equipment (MTS Systems Co., Eden Prairie, MN, USA) with a load capacity of 50 kN. The maximum testing frequency was ≤70 Hz. Tests were performed in force-control mode with active peak-value compensation to guarantee at least ±1% peak value accuracy.

### 2.3. Specimen Geometry

Specimen geometries are constrained mainly by the resonance criterion at approximately 20 kHz for ultrasonic fatigue tests, the self-heating properties of the investigated material, the maximum power, and achievable displacement amplitude of the used setup. Specimen geometries can be chosen more freely in conventional fatigue tests. However, if the comparability between conventional and ultrasonic fatigue tests is investigated, it is important to have a comparable material loaded under comparable conditions.

It is necessary that the loaded volume of specimens used in both testing series is identical. If a significantly larger testing volume would be used for conventional tests, for example, lifetimes would be shifted to lower values due to the size effect. It is, therefore, sensible to use the same gauge section geometry in low-frequency and ultrasonic tests to make the results comparable.

Besides the influence of testing volume, the stress distribution in the specimens must be considered. The specimen geometry can affect the stress distribution along the specimen’s length and over the load-bearing cross-section for conventional and ultrasonic resonance loading differently. Geometries featuring rectangular cross-sections, for example, exhibit a distinct stress profile across their cross-sections under resonance loading, where strain amplitudes can vary by as much as 10%. It is, therefore, necessary not only to exclude a size effect, but also to guarantee comparable stress distributions over the loaded cross-sections for low and ultrasonic tests.

**Figure 1:**
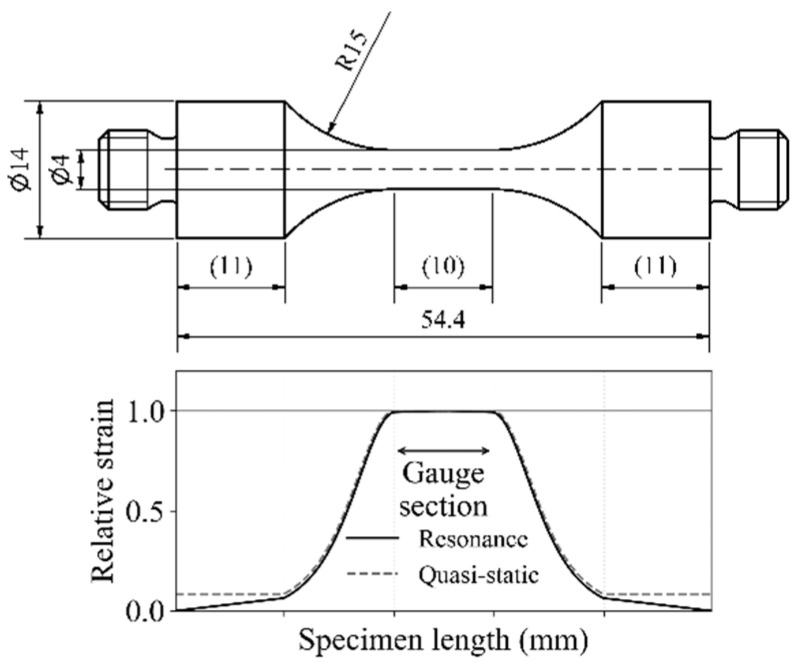
Specimen shape used for ultrasonic and servo-hydraulic fatigue tests (all dimensions in mm); strain distribution along the specimen’s length under ultrasonic resonance loading (solid line) and quasi-static (servo-hydraulic) loading (dashed line) is shown.

For the present study, authors tested fatigue performance of Ti6Al4V and 2024-T351 aluminium alloys using the same specimen geometry, as shown in Figure 1, by both servo-hydraulic and ultrasonic tests. The strain distribution along the specimen’s length was calculated with Autodesk Inventor Nastran 2021 with the *Normal Modes* and the *Linear Static* simulation types, respectively. A sufficiently high mesh resolution of 0.2 mm was chosen over the gauge section. Figure 1 shows that the cylindrical gauge section of 10 mm length experiences almost uniform strain (variation of strain amplitude is less than 1% in the modal, and 0% in the static loading case, respectively). Additionally, the stress distribution over the load-bearing cross-section (not shown) identifies less than 1% deviation from nominal stress for both loading conditions.

### 2.4. Materials

Ti6Al4V and 2024-T351 aluminium alloys were tested with displacement-controlled ultrasonic fatigue testing equipment in the range 19–20 kHz and with force-controlled servo-hydraulic or resonance fatigue tests at frequencies below 100 Hz.

The age-hardened aluminium alloy 2024 was tested under T351 conditions (i.e., solution annealed, water quenched, cold worked and age hardened). The chemical composition of the material was (in% mass): Cu 4.46, Mg 1.42, Mn 0.61, Zn 0.18, Si 0.06, Fe 0.14, Cr 0.005, Ti 0.025, Pb 0.0020, Ni 0.0058, Sn 0.055 and Al balance. The material was obtained in rolled plates. Mechanical properties are as follows: tensile strength 473 ± 3 MPa, 0.2% proof stress 364 ± 4 MPa, elongation 18 ± 1% and Vickers hardness 141 ± 2 HV.

Dumbbell-shaped specimens with cylindrical gauge sections were machined with their longitudinal dimension aligned to the rolling direction of the plates. The gauge sections were ground parallel to the specimen’s longitudinal dimension with abrasive paper of grade 1000 to obtain well-defined surface conditions. The same specimen shape was used for fatigue testing with ultrasonic and servo-hydraulic testing equipment. Specimen shape and strain distribution along the specimen’s length under servo-hydraulic and ultrasonic fatigue loading are shown in Figure 1.

The titanium alloy Ti6Al4V was tested in mill annealed condition. The chemical composition was (in% mass): Al 6.2, V 4.1, Fe 0.06, O 0.17, N 0.004, H 0.0013, C 0.014, Y < 0.001, other impurities total <0.04, Ti balance. Mechanical properties of the material are as follows: tensile strength 993 MPa, 0.2% proof stress 965 MPa, elongation 21% and Vickers hardness 331 HV.

Specimens were manufactured from rods with a diameter of 15 mm. The same specimen shape was used for testing Ti6Al4V as well as for 2024-T351 (Figure 1). The surface in the gauge section is ground parallel to the specimen’s longitudinal dimension with elastic grinding wheels of grade 150 (approximately equivalent to abrasive paper grade 1000). The same specimen geometry with the same surface preparation was used for servo-hydraulic and ultrasonic tests.

Fatigue tests were performed with thin sheets made of 18Ni maraging steel with nitrided surfaces [16]. The chemical composition was (in% mass): Ni 18, Co 5, Mo 5, Al 1, Cr 1 and Fe (balance). Sheet specimens were manufactured from coil material in solution-annealed condition, followed by precipitation hardening (480 °C, 2.5 h) and gas nitriding. The material showed a tensile strength of 2000 MPa and a yield strength of 1800 MPa. Vickers hardness of the nitrided surface layer was 1000 HV and 560 HV in the core of the sheet.

Two different specimen shapes were used in the tests, which are named large specimens and small specimens in the following [16]: The thickness of both specimens was 0.435 mm. Large specimens had constant cross-sections in the centre with a width of 14 mm and a length of 20 mm with a 170 mm shoulder fillet radius. The small specimens had a width of 2.4 mm in the centre. The width increased towards both ends with a radius of 20 mm. The maximum stress in small specimens was solely in the centre, whereas it was constant over a length of 20 mm in large specimens. Consequently, the stressed volumes were different. The material volume subjected to more than 95% of the nominal stress was 3.4 mm^3^ for the small specimens and 260 mm^3^ for the large specimens i.e., the testing volume was larger by a factor of 76 for the large specimens.

## 3. Conventional and Ultrasonic Fatigue Testing

### 3.1. Aluminium Alloys

Most aluminium alloys do not show a fatigue limit and they fail even at very high numbers of cycles [17]. However, for some cast aluminium alloys, a fatigue limit is observed. At stress amplitudes below the fatigue limit, fatigue cracks can initiate at casting porosity in these materials; however, the cracks are non-propagating and do not lead to fracture even if cycled to the VHCF regime [18,19]. In automotive applications, engine components, wheels and chassis components may be stressed with several hundred million cycles during service. The fatigue behaviour in the VHCF regime cannot be reliably extrapolated from HCF data, and accelerated testing techniques are therefore of great interest. Different types of aluminium-based alloys have already been investigated with ultrasonic fatigue testing, including wrought alloys, cast alloys, metal matrix composites, foams, laminates and additively manufactured (AM) alloys. Due to the low damping properties of most aluminium alloys, heat generation is low and fatigue tests can be performed with high effective frequency (i.e., test frequency considering that the specimen is not loaded continuously but in pulsed mode). Therefore, ultrasonic fatigue investigations up to 10^9^ cycles or beyond are possible within one or a few days.

**Figure 2:**
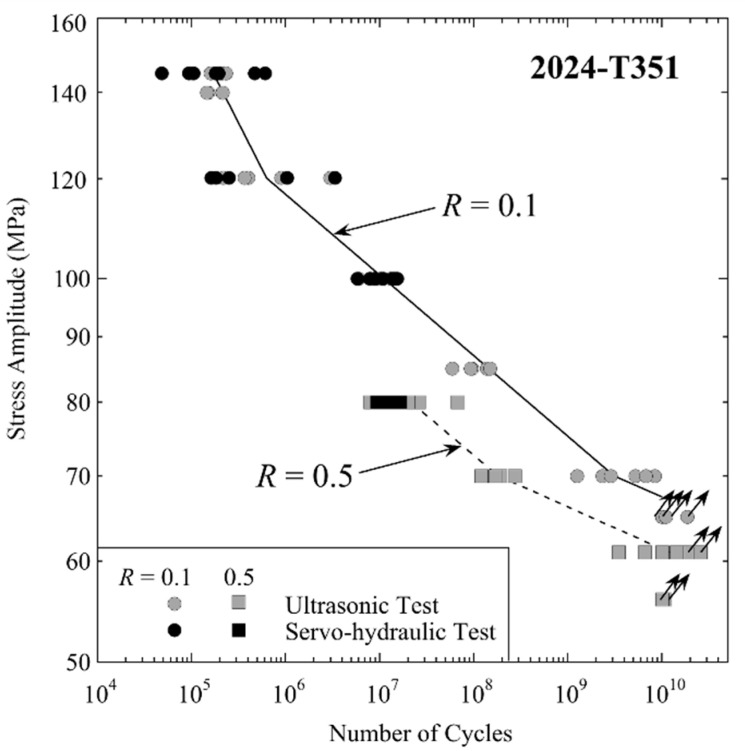
Fatigue data of aluminium alloy 2024-T351 measured at load ratio *R* = 0.1 (circles) and *R* = 0.5 (squares) with servo-hydraulic equipment at cycling frequencies between 8 Hz and 70 Hz (black symbols) and with ultrasonic equipment at 20 kHz (grey symbols).

Fatigue lifetimes for 2024-T351 in the HCF and VHCF regimes have been the subject of several investigations in the author’s laboratory, including constant and variable amplitude loading at different load ratios at low and ultrasonic frequencies [20,21,22] as well as ultrasonic cyclic torsion tests [23]. Fatigue performance of 2024-T351 specimens tested at *R* = 0.1 and 0.5 load ratios with both displacement-controlled ultrasonic testing equipment and with force-controlled servo-hydraulic testing equipment, is shown in Figure 2. Specimens with the shape shown in Figure 1 from the same batch and with the same surface preparation were used in both testing series. Tests with servo-hydraulic equipment were performed at cycling frequencies between 8 Hz and 70 Hz and ultrasonic tests at 20 kHz. Mean lifetimes measured at 20 kHz at *R* = 0.1 were 1.7 × 10^5^ cycles at 145 MPa, 6.7 × 10^5^ cycles at 120 MPa, and 1.1 × 10^7^ cycles at 100 MPa. In servo-hydraulic tests, mean lifetimes measured at these stress amplitudes were 1.6 × 10^5^ cycles, 4.8 × 10^5^ cycles and 1.0 × 10^7^ cycles. At load ratio 0.5 and stress amplitude 80 MPa, the mean lifetime is 1.2 × 10^7^ cycles at low and 2.2 × 10^7^ cycles at ultrasonic frequencies.

Considering all four testing series, the mean fatigue lifetimes were a factor 1.3 higher in ultrasonic than in servo-hydraulic tests. However, this must be compared to the scatter of fatigue lifetimes. Assuming a log-normal distribution of cycles to failure, lifetimes for 90% failure probability were factor 4 higher than those for 10% fracture probability for ultrasonic and servo-hydraulic tests. Thus, the deviation of mean lifetimes measured in ultrasonic and servo-hydraulic tests was much smaller than the scatter of lifetimes, and no statistically significant deviation of lifetimes was found.

**Figure 3:**
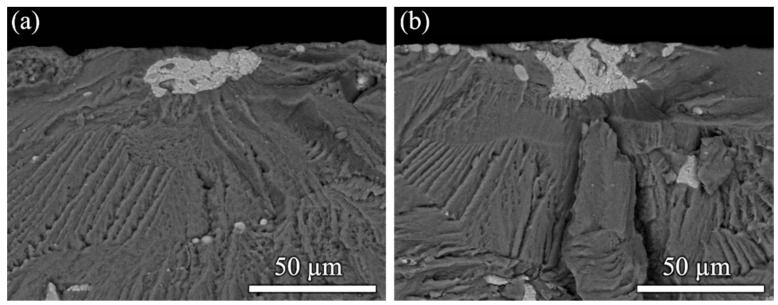
Fatigue crack initiation in 2024-T351 at a fractured secondary phase particle, probably Al_7_Cu_2_(Fe, Mn), cycling at stress amplitude 145 MPa (**a**) in ultrasonic test (1.8 × 10^5^ cycles to failure) and (**b**) in servo-hydraulic test (9.1 × 10^4^ cycles to failure).

Fracture surfaces at the places of fatigue crack initiation after fracturing at 145 MPa in the ultrasonic test and in the servo-hydraulic test are shown in Figure 3a,b, respectively. Cycles to failure were 9.1 × 10^4^ cycles in the test at low frequency and 1.8 × 10^5^ cycles in the ultrasonic frequency test. Images were obtained in backscatter electron mode to improve material contrast. Both figures show crack initiation at fractured secondary phase particles. Energy-dispersive X-ray analysis suggests that these are Al_7_Cu_2_(Fe, Mn) particles. Crack growth close to the particle was trans-granular, mainly quasi-cleavage fracture with a brittle appearance of the fracture surfaces. No obvious difference was found for crack initiation at low and ultrasonic frequencies indicating that the mechanism of fatigue damage is not influenced by the drastic increase of cycling frequency.

A wrought aluminium alloy similar to 7075 (i.e., alloy AlZnMgCu1.5) was tested with displacement controlled ultrasonic testing equipment and with a resonant testing machine working at 100 Hz [24]. Fatigue data in the regime from 10^5^ to 10^7^ cycles were measured at both frequencies, and comparable lifetimes were found.

Investigation of the fatigue performance of cast aluminium alloy E319-T7 with ultrasonic and servo-hydraulic equipment by Zhu et al. [9] delivered a different result. The measurements were also performed with displacement controlled ultrasonic fatigue testing equipment, and specimens with comparable geometry and stress distribution were used in tests at 20 kHz and 75 Hz.

**Figure 4:**
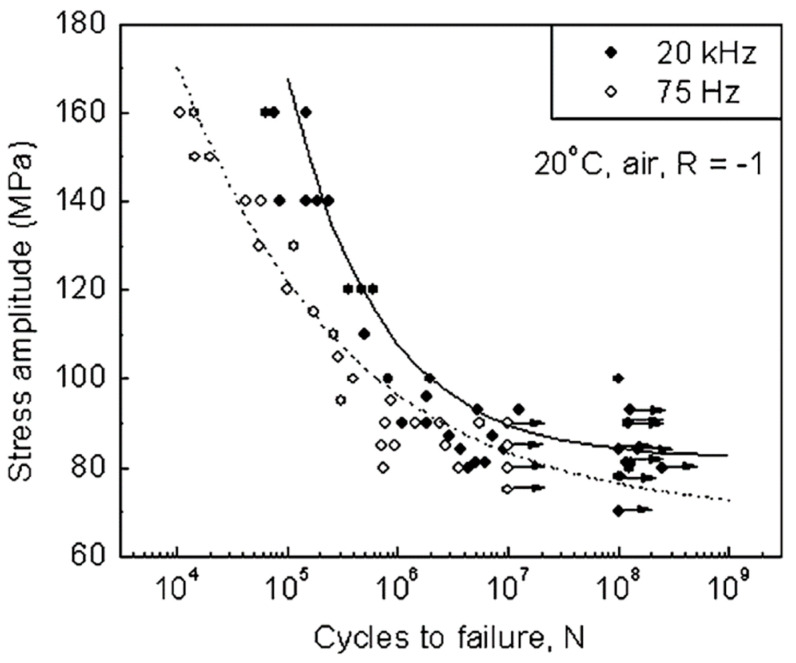
Fatigue data of cast aluminium E319-T7 with servo-hydraulic equipment at 75 Hz and with ultrasonic equipment at 20 kHz; reprinted with permission from Springer Nature [9].

The tests were performed at 20 °C, 150 °C and 250 °C, and results obtained at room temperature are shown in Figure 4. The mean number of cycles to failure was increased by a factor of 5 to 10 in ultrasonic tests at all three temperatures. The extended lifetimes in ultrasonic tests are explained by the environmental influence of air humidity, which is weaker at ultrasonic than at low cycling frequency. Fatigue loading leads to early crack initiation at porosity in the cast alloy. Water vapour of ambient air diffuses to the crack tip and causes material embrittlement. At ultrasonic frequencies, the crack is not open long enough for full coverage. Thus, the chemical processes caused by water vapour act more severely in the servo-hydraulic than in the ultrasonic test, and lifetimes are prolonged. Performing ultrasonic fatigue tests in distilled water significantly decreased the lifetimes and led to an *S-N* curve close to the *S-N* curve at 75 Hz in ambient air [10].

Caton et al. [19] investigated cyclic properties of W319-T7 cast aluminium alloy using displacement controlled ultrasonic equipment and compared these results with servo-hydraulic tests. Microstructures with different secondary dendrite arm spacings were studied. In the range of 10^5^ to 10^7^ cycles, fatigue data were produced at 40 Hz and 20 kHz cycling frequency and no frequency influence on fatigue lifetime was found. An interesting result, which could have solely been found with ultrasonic fatigue testing, was the demonstration of a fatigue limit in this cast aluminium.

The fatigue properties of Al1Mg0.6Si foam cylinders were tested at 1–10 Hz with a servo-hydraulic testing machine in the low and high cycle fatigue regime, and at 20 kHz using ultrasonic equipment in the HCF and VHCF regimes [25]. Fatigue date in the overlapping regime showed no significant influence of frequency and testing procedure. The foam shows an endurance limit, where cracks initiated internally in the closed-cell structure, at holes or pre-existing cracks, but these cracks did not propagate to fracture.

The powder metallurgical aluminium–silicon alloy DISPAL^®^ S232-T6x was tested with displacement controlled ultrasonic equipment at the authors’ laboratory at 20 °C and 150 °C [26]. In parallel, servo-hydraulic fatigue tests were performed at IWK Aachen, Germany. In the HCF regime, where fatigue lifetimes were measured with both methods, no influence of the cycling frequency was found at room or elevated temperature. Additional ultrasonic fatigue tests in distilled water delivered similar lifetimes to the ultrasonic tests in ambient air.

Additive manufactured (AM) AlSi10Mg produced by selective laser melting were studied by Awd et al. [27] with servo-hydraulic equipment at 20 Hz and with ultrasonic equipment at 20 kHz. They concluded that the influence of testing at ultrasonic frequencies on the measured fatigue data was not significant. Pores were found as preferential crack initiation locations at low and high cycling frequencies. Due to the possibility for rapid generation of HCF and VHCF data, the ultrasonic method is a powerful method to study AM aluminium alloys and was used to rate different alloys [28] and to investigate the influence of process parameters on cyclic strength [29].

Investigations of aluminium alloys show comparable lifetimes in ultrasonic and servo-hydraulic tests for some alloys, whereas others show prolonged lifetimes at high frequencies. Face-centred cubic (fcc) lattice structures are relatively insensitive to strain rate influences [7] which makes strain rate influences in aluminium alloys improbable. This is supported by fatigue crack growth investigations in a vacuum, where similar growth rates and threshold stress intensity factors were found at 20 Hz and 20 kHz [30,31]. In ambient air, humidity causes chemical processes at the crack tip that accelerate crack growth and deteriorate the cyclic properties. The ratio of water vapour partial pressure and cycling frequency, the *p*/*f* determines the diffusion rate of water vapour to the crack tip and consequently its deleterious effect. The diffusion rate is too low to allow full coverage of newly created surfaces with aluminium-hydroxide during an ultrasonic cycle [9,10], whereas this is possible for ambient air conditions and conventional cycling frequencies [32,33]. Increasing the water vapour pressure in ultrasonic experiments decreases the difference measured in low and ultrasonic frequency tests. Water partial pressure can be increased by a factor of about 1000, cycling the specimen in (distilled) water instead of humid air, i.e., the *p*/*f* for cycling a specimen at ultrasonic frequencies in water is in the same regime as the *p/f* for cycling a specimen at low frequency in humid air [9,34]. Experiments in water can, therefore, be used to assess whether an alloy is sensitive to air humidity, and frequency effects are possible or if the alloy is insensitive and ultrasonic tests and servo-hydraulic tests deliver comparable lifetimes.

### 3.2. Steels

**Figure 5:**
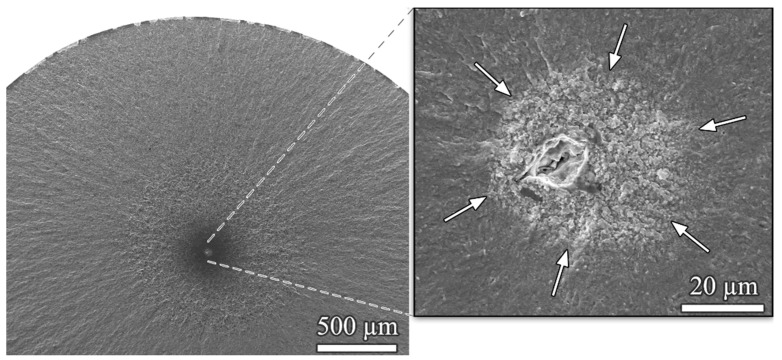
Fatigue crack initiation in 100Cr6 bearing steel at an interior non-metallic inclusion after ultrasonic fatigue testing at *σ*_a_ = 875 MPa, *R* = −1, *N*_f_ = 2.4 × 10^9^ cycles; the border of FGA is marked with arrows.

The failure of high-strength steels beyond the conventional fatigue limit at 10^7^ cycles was one of the main findings that triggered interest in the very high cycle fatigue (VHCF) regime [35,36,37]. Fatigue cracks preferentially initiate at the surface in the HCF regime whereas internal inclusions are a starting point for cracks leading to VHCF failure. In the latter case, the fracture surface in the close vicinity of the crack initiating inclusion shows a typical rough appearance, referred to as the optically dark area (ODA) [38,39] or fine granular area (FGA) [40] in tests performed at negative and slightly positive load ratios (i.e., *R* = 0.1). An example of VHCF failure from an interior non-metallic inclusion with a distinct FGA is shown in Figure 5. Crack propagation rates inside the FGA are two or more decades below one Burgers vector per cycle and nearly exclusively determine VHCF lifetime, and failures can be found up to extremely high numbers of cycles such as >2 × 10^10^ cycles [41]. Since such high numbers of cycles can only be reached with a high-frequency testing technique, ultrasonic fatigue has become a widely used testing technique for VHCF investigations of high-strength steels.

**Figure 6:**
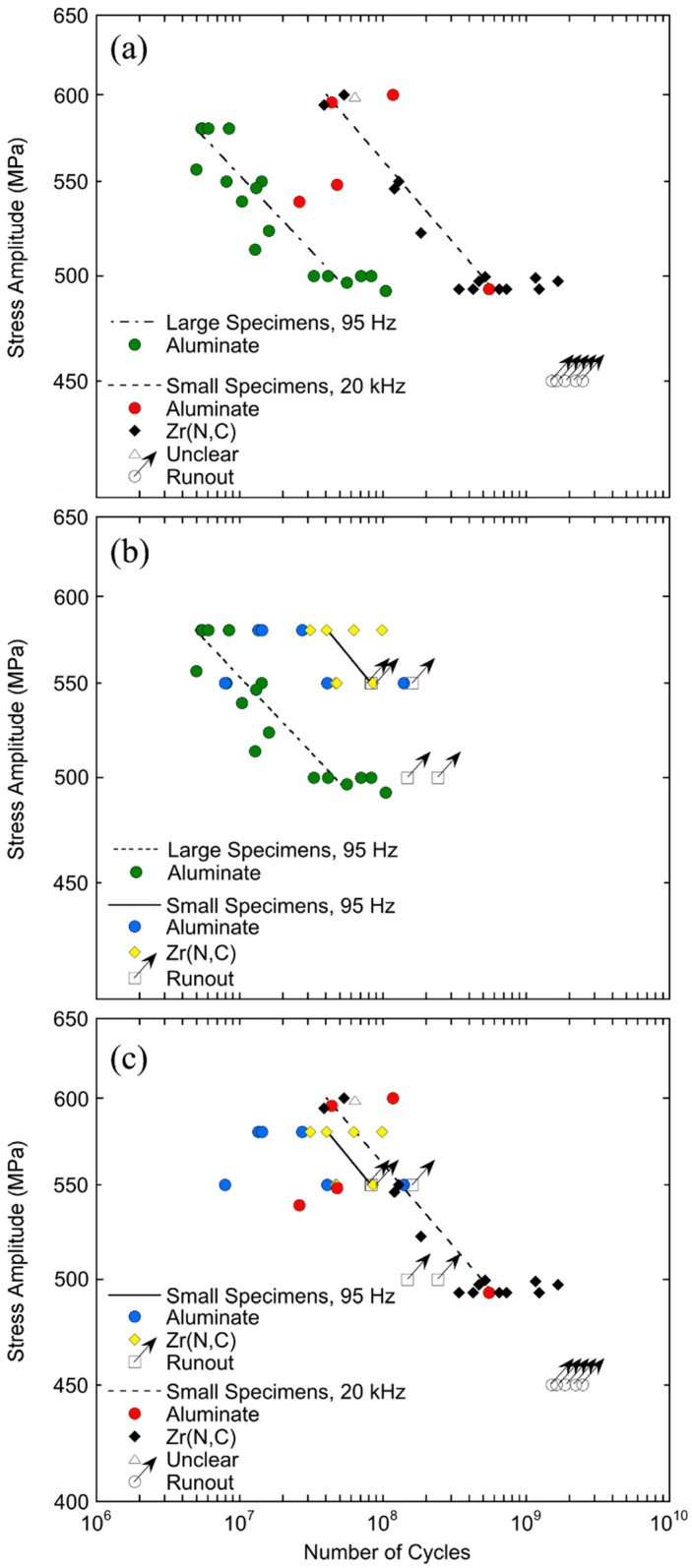
Comparison of *S-N* data at load ratio *R* = 0.1 (**a**) of large specimens tested at 95 Hz and of small specimens tested at 20 kHz; (**b**) of large and small specimens tested at 95 Hz; (**c**) of small specimens tested at 95 Hz and 20 kHz; crack initiation was at internal aluminate inclusions (circles) or at internal Zr(N,C) inclusions (diamonds); runouts are marked with arrows; reprinted with permission from Elsevier [16].

An investigation into frequency influences on fatigue properties of high-strength steels was performed with 18Ni maraging steel [16]. Figure 6 shows fatigue data of maraging steel sheet specimens obtained in three testing series: large specimens (testing volume 260 mm^3^) tested at 95 Hz, small specimens (testing volume 3.4 mm^3^) tested at 95 Hz, and small specimens tested at 20 kHz.

The result of the investigation shows that there is a strong size effect in this maraging steel. Mean lifetimes measured with large specimens are about one order of magnitude lower than mean lifetimes of small specimens. This is found when cycling the small specimen at ultrasonic frequencies (Figure 6a) or cycling both specimens at the same low frequencies (Figure 6b). In contrast, when cycling small specimens at low and ultrasonic frequencies, the difference in mean lifetimes is within the range of scatter (Figure 6c). The mean size of inclusions in large specimens is 9.9 µm, whereas it is 4.4 µm in small specimens. For the fatigue crack initiating at an internal inclusion, this means that the starting crack length was longer in large specimens, which shortened the crack propagation period and decreased the fatigue lifetime.

Cracks initiated exclusively at aluminates in large specimens. Crack initiation was at aluminate and Zr(N,C)-inclusions in small specimens for cycling with both 95 Hz and 20 kHz. Fracture of the interface between aluminates and matrix and particle fracture of the (smaller) Zr(N,C)-inclusions are the two crack initiating mechanisms. Due to the small highly stressed volume in small specimens, about half of the samples do not contain aluminate inclusions large enough to initiate a crack, and failure is caused by the smaller Zr(N,C) inclusions.

**Figure 7:**
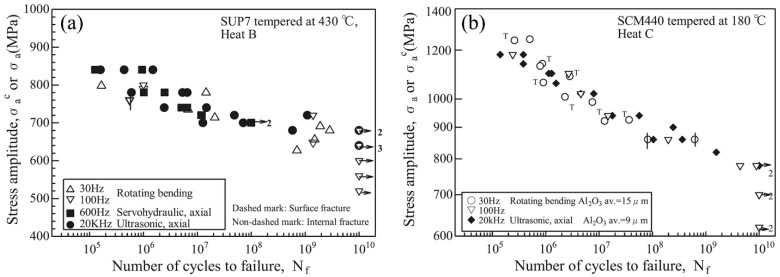
Fatigue data of high-strength spring steel SUP7 (**a**) and low-alloy steel SCM440 (**b**) with rotating-bending equipment (30 Hz and 100 Hz), servo-hydraulic equipment (600 Hz), and ultrasonic equipment at 20 kHz [42].

Japanese Researchers from the National Institute of Materials Science performed ultrasonic fatigue and multiple-axis, cantilever-type, rotating bending fatigue testing [42], up to 10^10^ cycles which took more than 3 years at 100 Hz. No frequency effects were found for the two investigated high-strength steels (spring steel SUP7 and low-alloy steel SCM440) as shown in Figure 7. The same research group further demonstrated that the size of the specimens significantly affects the fatigue lifetimes even in the VHCF regime [43,44] due to a lower probability of large inclusions in small testing volumes—as discussed for 18Ni maraging steel sheets.

In addition to the examples demonstrated above, several investigations of the VHCF properties of martensitic stainless steels [41,45,46,47] have shown that ultrasonic frequencies do not affect the fatigue lifetimes if the failure occurs due to inherent defects such as non-metallic inclusions.

**Figure 8:**
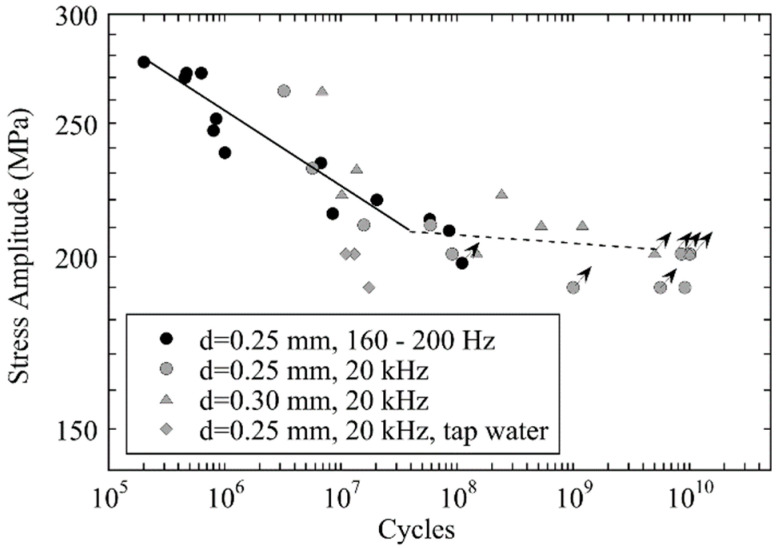
Fatigue data of tubes made of austenitic stainless steel AISI 904L measured with rotating bending at 160 Hz–200 Hz and ultrasonic fatigue equipment at 20 kHz; adapted with permission from John Wiley and Sons [49].

In contrast to martensitic steels, where the inclusion size is large compared to the characteristic microstructural size (block size) [48], austenitic steels may rather fail due to crack initiation at surface slip bands. In this case, it can be assumed that the austenite grain size determines the fatigue strength. Bending fatigue tests at 160 Hz to 200 Hz and ultrasonic fatigue tests at 20 kHz were performed with tubular specimens made of austenitic stainless steel AISI904L by Carstensen et al. [49] (Figure 8), where no frequency effect was observed. Ultrasonic and resonance pulsating fatigue tests at 150 Hz were also performed with austenitic stainless steel AISI316L steel [50]. A significantly increased fatigue limit and prolonged fatigue lifetimes were observed at 20 kHz. The strong damping property of austenitic steel required to use specimens with much smaller testing volume in ultrasonic tests, and the difference in measured strength was attributed to frequency as well as scale effects [50].

**Figure 9:**
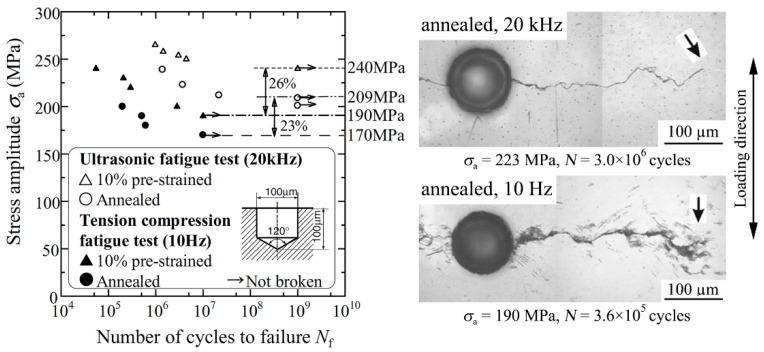
Servo-hydraulic and ultrasonic tension-compressions fatigue test results with annealed and 10% pre-strained low carbon steel specimens containing 1-hole defects; reprinted with permission from John Wiley and Sons [52].

In contrast to the fcc austenite, it is well known that a body-centred cubic (bcc) ferritic microstructure exhibits strain-rate dependent mechanical properties [7,8]. Not only yield and tensile strength increase with a higher strain rate [51] but also the fatigue properties are exaggerated at higher testing frequencies. Frequency effects in low carbon steels, where fatigue crack initiation occurs in the ferrite phase, were systematically investigated by Tsutsumi et al. [52] employing conventional tension-compression tests at 10 Hz and ultrasonic-fatigue testing. As shown in Figure 9, fatigue lifetimes and fatigue limit were increased at 20 kHz for both annealed (Vickers hardness of *HV* = 123) and 10% pre-strained (*HV* = 160) specimens. This was explained by larger cyclic yield stress and a reduction in plastic zone size at the crack tip in ultrasonic fatigue tests [52]. The latter was corroborated by the observation of abundant slip bands around fatigue cracks under conventional fatigue tests that were rarely visible after ultrasonic fatigue testing (Figure 9).

Extended fatigue lifetimes in ultrasonic fatigue tests were reported in several investigations of mild steels [53,54,55], and the strain rate sensitivity of ferrite, decreased plastic deformation at high strain rates and retardation in crack formation in the bcc phase are held responsible. Attempts have been made to consider the strain rate sensitivity of ferrite in the presentation of ultrasonic fatigue data. It has been shown [55] that if lifetimes are presented versus normalised stress amplitudes, i.e., the ratio of stress amplitude and tensile strength at room temperature (conventional testing) and the ratio of stress amplitude and tensile strength at 350 °C (ultrasonic testing), low and high-frequency data show good agreement. Other investigations [56,57] have considered ways to control the fatigue tests, i.e., that ultrasonic experiments are displacement controlled whereas servo-hydraulic tests are force controlled. Considering the strain rate dependence of yield strength, ultrasonic stresses are re-calculated using the cyclic stress–strain curve, which then leads to a good agreement between conventional and ultrasonic fatigue data.

**Figure 10:**
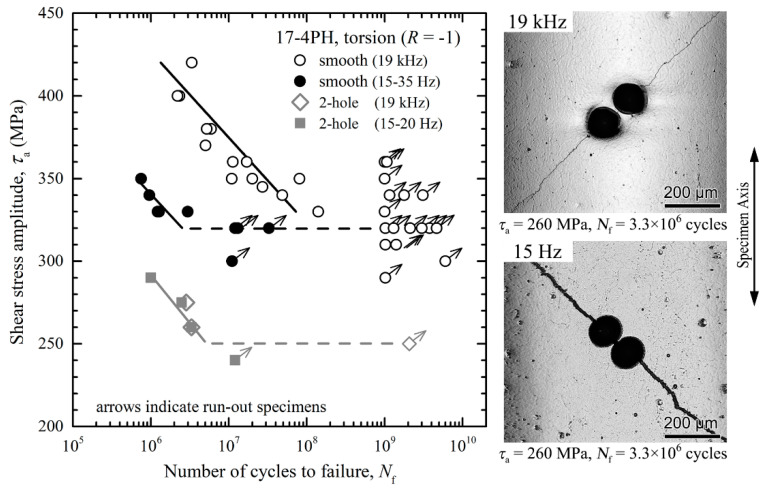
Servo-hydraulic and ultrasonic torsional fatigue tests with martensitic stainless steel 17-4PH (smooth and 2-hole defect containing specimens) [59].

Surprising observations were recently made with the martensitic stainless steels 17-4PH: while no frequency effects were apparent from the results obtained by servo-hydraulic tension-compression, rotating-bending or ultrasonic fatigue testing [41,58], fatigue lifetimes were significantly prolonged under cyclic torsional loading at ultrasonic frequencies [59] (Figure 10). This can be explained by the presence of elongated δ ferrite grains where shear cracks can more easily initiate under low-frequency cycling due to the strain-rate sensitivity of the bcc microstructure. Under uniaxial and rotating bending loading, δ ferrite grains were oriented parallel to the maximum principal stress direction. In this case, the relevant grain size is similar to the diameter of non-metallic inclusions, and failure was more likely to occur from the latter [41], regardless of the testing frequency. Under torsional loading, however, δ ferrite grains were inclined by approximately 45° to the direction of the major principal stress and, thus, were oriented parallel to the maximum shear direction. This facilitated the initiation of shear cracks with a length exceeding the size of inherent defects, such as inclusions of uncritical size. As shown in Figure 10, the influence of testing frequency under torsional loading disappeared when artificial defects larger than a critical size were introduced in the test specimens.

Based on these research results it can be concluded that frequency effects can be neglected if non-metallic inclusions or other defects are the origins of the fatigue fracture. Small defects are the source of the fatigue fracture in high-strength steels and determine their cyclic strength [60]. Several ultrasonic and conventional investigations of the HCF and the VHCF regimes have shown the absence of a frequency effect. A strong size effect due to the increasing probability of encountering large defects with increasing testing volumes must be considered when comparing fatigue data measured with different methods. If the fatigue cracks in steels initiate in a ferritic phase, however, higher fatigue limits and lifetimes are observed in ultrasonic fatigue experiments due to the strain-rate sensitivity of the bcc microstructure. This leads to reduced plastic deformation, a prolonged crack initiation period and an extended crack propagation period. In special cases, this effect may even be relevant in high-strength steels, for example, if ferritic grains are embedded in a martensitic matrix and if they are of sufficient size in the maximum shear direction. In austenitic stainless steels, no frequency effects are expected due to the fcc microstructure. However, comparative tests with conventional and ultrasonic fatigue tests with austenitic steels are limited, and further investigations are necessary.

### 3.3. Titanium Alloys

Titanium alloys are commonly used in aerospace industries, where their high strength and relatively low weight allow for significant weight savings. The additional high corrosion resistance makes them favourable for applications where good performance under high frequencies and at high numbers of load cycles regime is required. HCF-related gas turbine engine failures observed by the US Air Force led to increased interest in fatigue of Ti6Al4V in the early 1990s, resulting in the “High Cycle Fatigue Science And Technology Program” by the US Air Force, starting in 1994 (e.g., [61]). Today, the interest in titanium alloys extends to additively manufactured (AM) applications, where a methodology for the rapid characterisation of fatigue properties for the many different processes, build conditions and post-processing procedures could greatly speed up material development.

**Figure 11:**
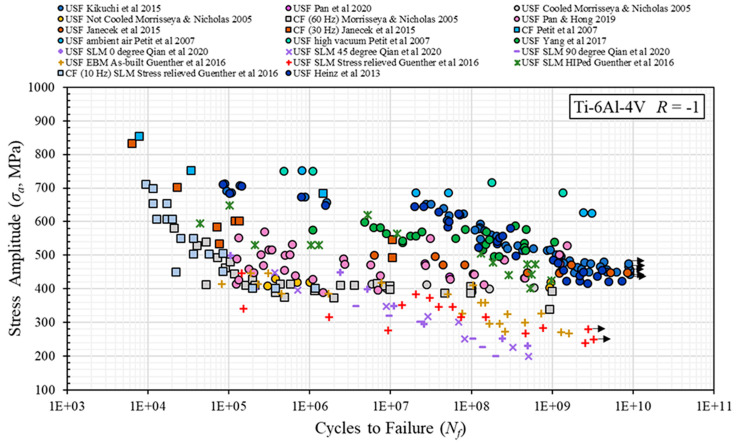
Fatigue data for Ti6Al4V across numerous investigations, generated by conventional and ultrasonic fatigue testing at *R* = −1 [62,63,64,65,66,67,68,69,70,71].

Several studies investigated the fatigue performance of Ti6Al4V at *R* = −1 with conventional and ultrasonic fatigue (Figure 11). Generally, wrought Ti6Al4V tested in a high vacuum showed the best performance followed by wrought Ti6Al4V tested in ambient air. The HIPed AM specimens had shown fatigue performance as good as the wrought material and even better than cast material. Finally, non-HIPed AM specimens showed the poorest fatigue performance. As-built electron beam melting (EBM) processed specimens and selective laser melting (SLM) processed and heat-treated specimens showed almost equal fatigue performance, since they have been shown to be similar in terms of defect sizes and distribution.

Most studies, however, only present either data produced with the conventional testing technique or data from ultrasonic tests, respectively (Figure 11). Direct comparison between conventional and ultrasonic fatigue data across different investigations is not possible, due to the large number of stock material conditions (cast, wrought, AM), heat treatments (affecting, e.g., primary α grain sizes), testing methodologies and specimen geometries. This makes it difficult to draw meaningful conclusions regarding only the effect of testing frequency. It is necessary to test material of the same lot with the same gauge section geometry in both conventional and ultrasonic equipment to only look at the effect of test frequency.

**Figure 12:**
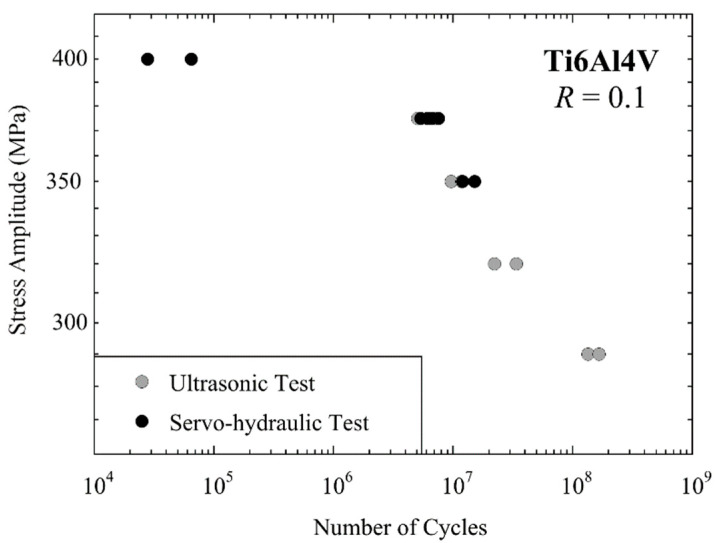
Fatigue data of Ti6Al4V ELI (mill annealed) measured at *R* = 0.1 with servo-hydraulic equipment at 55 Hz cycling frequency (black) and with ultrasonic equipment at 20 kHz (grey).

The authors measured the fatigue performance of mill annealed Ti6Al4V using both servo-hydraulic and displacement-controlled ultrasonic fatigue testing equipment. Specimens from the same stock with identical geometry (Figure 1) and identical surface preparation were fatigued in ambient air (24 °C, RH 50%) at *R* = 0.1 at 55 Hz cycling frequency (servo-hydraulic, force-controlled) and ~20 kHz (ultrasonic, displacement controlled), respectively. The resulting lifetimes are shown in Figure 12. Nine samples were cycled with servo-hydraulic equipment with stress amplitude, *σ*_a_ between 350 MPa and 400 MPa, and eight samples with ultrasonic equipment between 290 MPa and 375 MPa. For 350 MPa and 375 MPa, lifetimes measured with both methods are available. Measured lifetimes were very similar, i.e., the mean lifetimes only deviate by factor 1.1. Assuming a log-normal distribution of cycles to failure, lifetimes for 90% failure probability were a factor 2.2 higher than for 10% fracture probability for ultrasonic and servo-hydraulic tests. Approximating data with a power fit, a mean lifetime of 10^7^ cycles was found at *σ*_a_ = 359.7 MPa for servo-hydraulic and *σ*_a_ = 356.3 MPa for ultrasonic tests, respectively, i.e., the difference is less than 1%. Thus, the deviation of mean lifetimes measured in ultrasonic and servo-hydraulic tests is much smaller than the scatter of lifetimes, and no statistically significant deviation of lifetimes is found.

**Figure 13:**
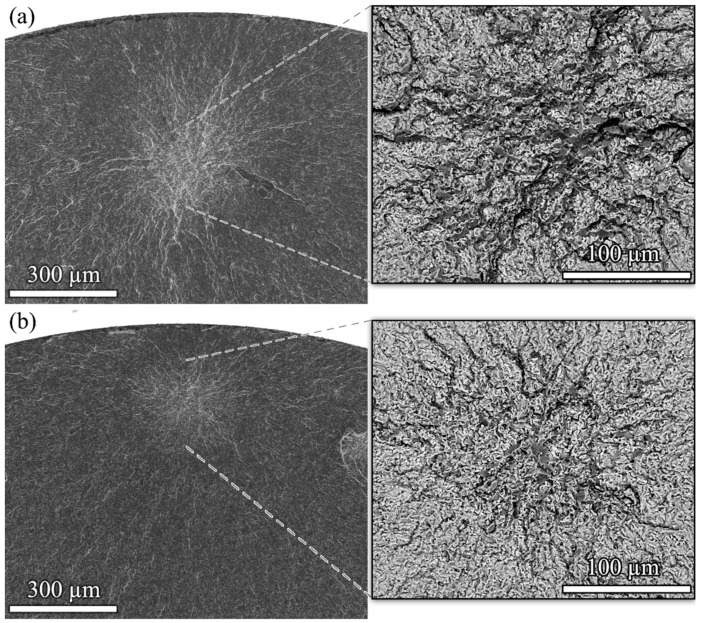
Fracture surfaces from specimens that were cycled at *σ*_a_ = 350 MPa; at (**a**) 20 kHz, *N*_f_ = 1.18 × 10^7^ and at (**b**) 55 Hz, *N*_f_ = 1.51 × 10^7^.

The fractographic investigation confirmed similar surface morphologies for both testing series: Figure 13 shows fracture surfaces of specimens that were cycled at *σ*_a_ = 350 MPa and that failed at approximately 10^7^ cycles. Both consistently show agglomerations of primary α grains at the crack initiation locations in the back-scatter images (right column). There the material is weakened due to the softer α phase, and cracks are preferentially initiated.

The Ti6Al4V fatigue data from the literature verify these findings. Morrissey and Nicholas [63,72] performed fully reversed fatigue tests with Ti6Al4V (tensile strength 968 MPa) with servo-hydraulic equipment at 60 Hz and with ultrasonic equipment at 20 kHz. *S-N* data are available from both testing methods in a wide range between 3 × 10^5^ and 10^8^ cycles. Very consistent lifetimes were found at both frequencies, indicating that no frequency influence is present in the investigated conditions.

Similarly, Takeuchi et al. [73] tested Ti6Al4V from three different manufacturers (tensile strengths between 906 MPa and 967 MPa; all three heats satisfied ASTM specifications) at 120 Hz, 600 Hz and 20 kHz at *R* = −1. Two of the three heats showed internal crack initiation above approximately 5 × 10^6^ cycles. Fatigue lifetimes measured for these two heats showing internal crack initiation in the long lifetime regime were similar for all three testing frequencies. However, the third heat showed solely surface crack initiation and no failures above 5 × 10^6^ cycles as well as the significant effect of the testing method and significantly higher cyclic strength when tested at the higher frequencies.

Furuya and Takeuchi [74] studied Ti6Al4V from the same three suppliers at *R* = 0 and *R* = 0.3 as well as with fixed maximum stress and cycling frequencies of 120 Hz and 20 kHz. With superimposed tensile mean loads, all three heats showed failures above 5 × 10^6^ cycles with crack initiation in the interior. No effect of the cycling frequency was found in the HCF and VHCF regimes for nine testing series overall.

Günther et al. [67] investigated the impact of EBM and SLM additive manufacturing on the fatigue life of Ti6Al4V in the HCF and VHCF regimes. Cylindrical fatigue specimens in differently post-treated conditions were tested at 10 Hz with servo-hydraulic equipment and at 20 kHz using ultrasonic equipment. Data from the SLM batch shows that the lifetimes and failure modes (i.e., crack initiation locations) are closely similar for both testing methods.

The fatigue properties of laser-additive manufactured Ti6Al4V were investigated by Wycisk et al. [75]. Specimens in stress relieved as well as HIPed conditions were analysed for crack initiation site, mean stress sensitivity and overall fatigue performance. Fatigue tests in tension-compression loading at 59 Hz and 20 kHz in the HCF and VHCF regimes until 10^9^ cycles revealed a clear shift of crack initiation from surface to internal initiation with increasing cycles to failure, regardless of the testing principle. They also stated that no effects of test frequency on life span could be determined.

Other than Ti6Al4V, Papakyriacou et al. [76] tested Ti6Al7Nb that is used for medical applications, with rotating bending equipment at 100 Hz and with ultrasonic equipment at 20 kHz. Fatigue lifetimes between 10^5^ and 2 × 10^8^ cycles were measured with both methods, and no frequency effect on fatigue lifetimes was found for this alloy.

Szczepanski et al. [77] characterised the fatigue behaviour of the alpha-beta titanium alloy Ti6Al2Sn4Zr6Mo in the HCF regime using conventional testing at 20 Hz and in the VHCF regime using ultrasonic equipment at 20 kHz, respectively, at a load ratio of 0.05. The lifetimes they found in both testing series showed a clear separation between earlier failures with crack initiation at the surface and longer lifetimes with crack initiation occurring below the surface. They concluded that, even though the stress levels of the employed testing methods do not overlap, the data follow the same trend as expected for a typical *S-N* curve. Together with a similar fractographic appearance, the authors interpret this as an indication that there is no appreciable frequency effect on fatigue lifetime between testing frequencies of 20 kHz and 20 Hz in the investigated Ti6Al2Sn4Zr6Mo alloy.

These results show that titanium alloys may be considered insensitive to frequency effects. Similar lifetimes are found with conventional and ultrasonic equipment, and crack initiation occurs preferentially in the interior at α grains in the VHCF regime for both low and high-frequency testing. The absence of a frequency effect may be expected, irrespective of the way titanium alloys are produced. However, the actual production process of a given titanium alloy can strongly affect the microstructure and consequently the measured fatigue properties.

### 3.4. Nickel Alloys

Components made of nickel-based superalloys such as turbine blades are typically exposed to a very high number of load cycles, and investigations of the VHCF regime are, therefore, of major importance. In the absence of detrimental defects, e.g., process-related flaws, such as casting pores or lack of fusion, polycrystalline nickel-based alloys typically fail due to favourably oriented large grains located at the surface or in the interior [78,79].

**Figure 14:**
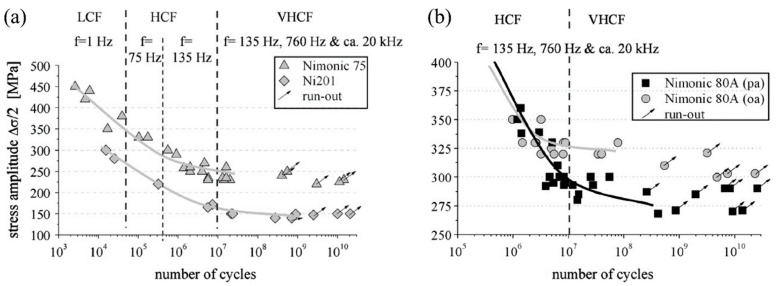
Fatigue data of different Ni-based alloys obtained with servo-hydraulic, resonance electromechanical and ultrasonic fatigue testing; (**a**) pure nickel (Ni201), Nimonic 75 and (**b**) peak-aged (pa) and overaged (oa) Nimonic 80A; reprinted with permission from Elsevier [80].

Stöcker et al. [80] performed fatigue tests with nickel alloys over a wide range of lifetimes, from low cycle fatigue to very high cycle fatigue, and obtained a single curve (Figure 14). Other investigations using ultrasonic fatigue testing similarly showed no frequency effect [81,82]. Due to their practical importance for gas turbine engines, the high-temperature fatigue properties of nickel alloys are important. This led to interesting further developments of the ultrasonic fatigue testing technique to be used up to 1000 °C [82,83].

### 3.5. Magnesium Alloys

**Figure 15:**
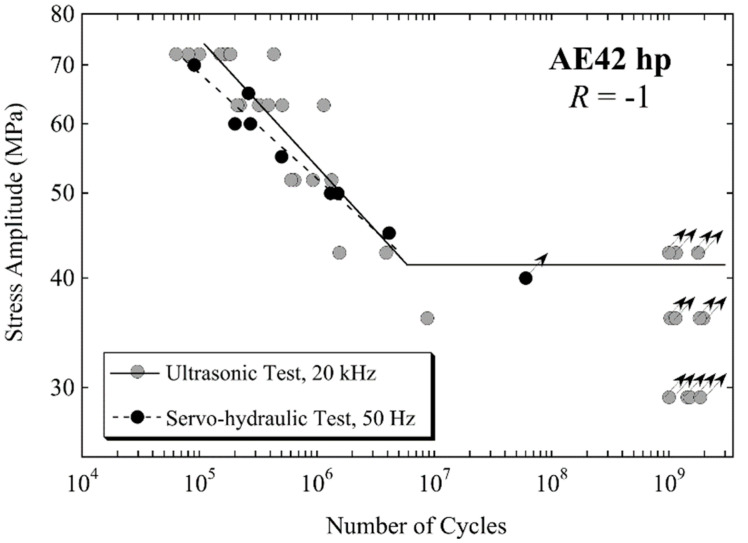
Fatigue data of magnesium alloy AE42 hp produced by high-pressure die-casting; data measured with servo-hydraulic (black circles) and ultrasonic equipment (grey circles) are shown; adapted with permission from Elsevier [18].

The three high-pressure die-cast magnesium alloys AZ91 hp, AM60 hp and AE42 hp, were investigated using ultrasonic equipment and servo-hydraulic equipment [18]. As an example, Figure 15 shows the fatigue data of AE42 hp measured with both methods. Fatigue cracks initiate at shrinkage and gas porosity in these high-pressure die-cast materials. Fatigue lifetimes show pronounced scatter due to the different sizes of crack-initiating defects in the different specimens. Within the ranges of scatter, similar lifetimes were measured for the three alloys at both 50 Hz and 20 kHz, respectively. Non-propagating cracks were found at porosities in runout specimens. Porosity can be considered an initial crack, and the fatigue limit can be correlated with the minimum stress intensity factor required to propagate the crack to fracture [18].

### 3.6. Fibre-Reinforced Polymers Composites

The number of applications of polymers and their fibre-reinforced composites has been growing rapidly during the past few decades, due to their low density, excellent chemical resistance, design flexibility, ease of manufacturing and robust mechanical properties. Such materials are used in the aerospace and automotive industries where components may be subjected to high numbers of load cycles. However, cyclic properties of fibre-reinforced polymer composites (FRPC) are mostly investigated in the regime below 10^6^ cycles. Except for ultrasonic studies, only one gigacycle fatigue investigation can be found in the literature where a carbon fibre-reinforced composite was tested with a resonance tester [84]. VHCF investigations with conventional methods are time-consuming, and therefore attempts have been made to use ultrasonic equipment for fatigue testing of FRPC. This is challenging due to the viscoelasticity of polymers and the associated heat generation at ultrasonic frequencies. Due to the low thermal conductivity coefficient of polymers (~0.2–0.5 W/m/K) compared to metals (~20–300 W/m/K), great care must be taken to avoid excessive heating during high-frequency cycling.

**Figure 16:**
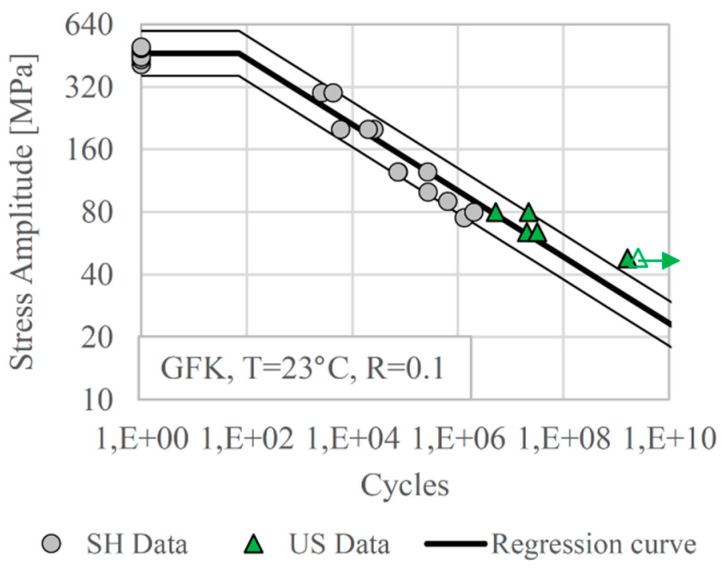
Fatigue data of a quasi-unidirectional glass fibre-reinforced polymer measured at load ratio *R* = 0.1 with servo-hydraulic equipment at 10 Hz and ultrasonic equipment at 20 kHz; reprinted with permission from Elsevier [85].

Testing glass fibre-reinforced polymer (GFRP) in pulsed mode, with forced air-cooling and with control of specimen surface temperature, it was shown that ultrasonic fatigue testing could be an appropriate technique for gathering valid fatigue data of FRPC [85]. Ultrasonic tests were performed with a static load superimposed to the resonance vibration to realise load ratio *R* = 0.1. Figure 16 shows the results of ultrasonic and servo-hydraulic fatigue tests of quasi-unidirectional GFRP (90% of the fibres aligned in the main direction while 10% of them are woven transversally for stabilisation) with a fibre volume fraction of 60%. Fatigue strengths measured with both techniques agree well, which indicates the suitability and reliability of the developed ultrasonic testing method.

Cyclic properties of GFRP were also studied by Lee et al. [86] using ultrasonic and hydraulic equipment working at 3 Hz, respectively. Tests were performed under fully reversed loading conditions, and shorter lifetimes were found in the ultrasonic tests. Since the results of ultrasonic tests are influenced by numerous parameters (e.g., calibration procedure, calculation of cyclic stress amplitude, the accuracy of equipment, the temperature of the specimen) and different specimen shapes were used in low and ultrasonic tests, a number of reasons for shorter lifetimes other than the frequency are possible.

Several ultrasonic studies showed the benefits of high-frequency testing for understanding the process of fatigue damage in FRPCs in the HCF and VHCF regimes. A three-point bending setup, where a specimen was stimulated to bending resonance vibrations at ultrasonic frequencies, was used by Backe et al. [12,87] to investigate the VHCF properties of carbon fibre-reinforced polymer (CFRP). These studies demonstrated the progress of fatigue damage as a multi-stage process starting with the debonding of fibre and matrix, then initiation of transverse cracks in the 90° layers, micro delamination between 0° and 90° fibre roving, followed by macro delamination and finally fracture. Cui et. al. [88] performed similar three-point bending ultrasonic tests of CFRP and showed differences in failure form in the low, high and very high cycle fatigue regime. Ding and Cheng studied the benefit of incorporating nano-silica [89] and multiwall carbon nanotubes [90] into carbon fibre-reinforced composites and reported great improvement in mechanical properties and fatigue resistance of composite materials in presence of nanoparticles.

### 3.7. Graphite

Fatigue properties of isotropic polycrystalline porous graphite (mean grain size 10 µm, porosity 13 vol%) were investigated in the regime between 10^3^ and 10^9^ cycles. Cyclic tension-compression tests with ultrasonic equipment at 20 kHz and fully-reversed cyclic bending tests at 25 Hz were performed [91]. The tensile strength of graphite is lower than its bending strength. Comparing the results of both testing series, this is considered by presenting lifetimes versus normalised stress amplitudes. The normalised stress amplitude is the ratio of tension-compression stress amplitude and tensile strength in ultrasonic tests, and the ratio of bending stress amplitude and bending strength in cyclic bending tests.

**Figure 17:**
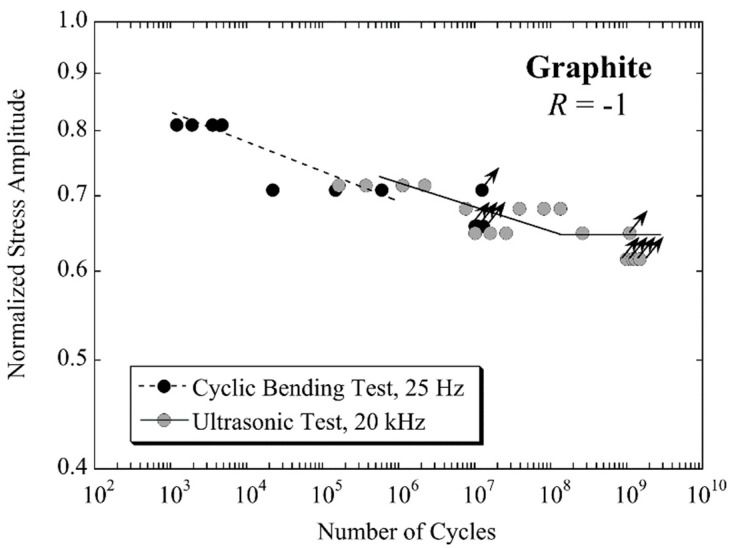
Fatigue data of polycrystalline graphite measured under fully reversed loading condition for cyclic bending (black circles) and ultrasonic tension–compression loading (grey circles), respectively; fatigue lifetimes are presented versus normalised stress amplitudes; adapted with permission from Elsevier [91].

Figure 17 shows fatigue lifetimes of graphite measured at 25 Hz and 20 kHz. Ultrasonic fatigue data are slightly shifted towards higher lifetimes compared with cyclic bending data; however, this difference is well within the range of scatter. It can be concluded, therefore, that the ultrasonic testing technique is appropriate for the rapid collection of fatigue data of graphite. Moreover, ultrasonic fatigue testing was also used to demonstrate the beneficial effect of infiltration of graphite with AlSi7Mg alloy cyclic strength [91].

### 3.8. Other Materials

In addition to the aforementioned material systems, several others have been successfully tested with ultrasonic equipment.

Fatigue crack initiation, slow crack propagation and conditions for crack arrest were studied in the aluminium laminates ARALL [92] and GLARE [93]. These materials are used in aerospace applications, where crack initiation at notches and slow fatigue crack growth are of great practical interest. Particularly, GLARE with glass fibres preloaded in the tension demonstrated strongly improved cyclic properties compared to aluminium alloy laminates [93].

In a specifically developed setup, thin wires (Ø0.1 mm) of the high-entropy alloy CoNiCr alloy MP35N were tested in the VHCF regime [94]. In this special application, the wire specimen does not vibrate in resonance, but is cyclically stressed in a quasi-static fashion at ultrasonic frequencies. The usability of the newly developed method was demonstrated by measuring lifetimes that are comparable to conventional tests [94].

Cyclic compression loading was purpose-developed for VHCF testing of concrete. With this technique, the damage mechanism in the very long lifetime regime could be demonstrated for concrete used in wind power plant foundations [95].

Fundamental studies on the fatigue mechanisms have been extensively performed with pure copper. It was shown, for example, that new persistent slip bands are formed even after 5 × 10^8^ cycles [96].

Phase transformation and associated fatigue damage were studied in the shape memory alloy Nitinol [97]. In situ synchrotron experiments successfully demonstrated the occurrence of the forward and reverse transformation between the material’s austenitic and martensitic phases in a super elastic state at ultrasonic frequencies.

## 4. Conclusions

Based on the literature and original data, this paper summarises the current understanding of whether and how fatigue properties measured with ultrasonic and conventional equipment are comparable.

- While the orders of magnitude are faster than conventional fatigue testing, no generally accepted standard for ultrasonic fatigue testing exists. The use of equipment with closed-loop control of vibration amplitude and the resonance frequency is strongly advised since this guarantees high accuracy and reproducibility of ultrasonic tests. Pulsed loading and appropriate cooling are necessary to avoid specimen heating.
- Depending on the material, frequency influences can be caused by strain rate influences on plastic deformation, and by time-dependent influences of the testing environment. The size effect must be considered for all materials, if low and ultrasonic frequency data are compared.
- Several aluminium alloys show comparable lifetimes in ultrasonic and conventional tests. Some alloys tested in ambient air show prolonged lifetimes at high frequency, due to a reduced influence of air humidity. Ultrasonic tests in high humidity or in distilled water can better approximate the environmental conditions acting at low cycling frequencies.
- Frequency effects can be neglected in high-strength steels, where non-metallic inclusions or other defects are preferential crack initiation locations in the regime of long lifetimes. Ultrasonic tests of steels lead to prolonged lifetimes if fatigue cracks initiate in a ferritic phase. Austenitic stainless steels are less prone to frequency effects.
- Titanium alloys may be considered insensitive to frequency effects, as suggested by data from both the original work and the literature. Similar lifetimes and similar crack initiation locations are found with conventional and ultrasonic equipment.
- Ultrasonic tests with nickel alloys showed no frequency effect. On the basis of limited data, the same conclusion can be drawn for cast magnesium alloys and graphite.
- Ultrasonic tests of a glass fibre-reinforced polymer delivered comparable fatigue lifetimes to servo-hydraulic tests, i.e., high-frequency testing is in principle applicable to testing fibre-reinforced polymer composites. However, further research is needed to better understand the influence of the experimental procedure on the measured data.

## Data Availability

The data are not publicly available as further investigations are currently ongoing.
